# Bibliometric Analysis of the Research on the Impact of Environmental Regulation on Green Technology Innovation Based on CiteSpace

**DOI:** 10.3390/ijerph192013273

**Published:** 2022-10-14

**Authors:** Mingxing Li, Xinxing Wang, Zihao Wang, Babar Maqbool, Abid Hussain, Waris Ali Khan

**Affiliations:** 1 School of Management, Jiangsu University, Zhenjiang 212013, China; 2 School of Medicine, Jiangsu University, Zhenjiang 212013, China; 3 Department of Veterinary Medicine, The University of Agriculture, Dera Isamil Khan 29050, Pakistan; 4 Department of Political Science, Bagdad ul Jadeed Campus, The Islamia University, Bahawalpur 63100, Pakistan; 5 Faculty of Business Economics and Accountancy, University Malaysia Sabah, Kota Kinabalu 88400, Sabah, Malaysia

**Keywords:** environmental regulation, green technology innovation, CiteSpace, bibliometrics

## Abstract

With increasing environmental regulation (ER), the requirements for green technology innovation (GTI) in enterprises are also rising. However, there are relatively few systematic summaries of the themes of ER-affecting GTI. Adopting the method of bibliometrics and visual analysis, this research discusses the status of research and development trends of ER-affecting GTI and summarizes the research in this field. The paper takes 738 papers from 2001 to 2021 in the core database of Web of Science as the research sample. Based on CiteSpace, this paper makes a visual analysis of the number of published papers, institutions, authors, keywords, countries (regions) and journals. The study found that to some extent, favorable collaboration between authors and institutions in this field needs to be strengthened. Research hotspots in this field include innovation, technology, performance, policy and environmental regulation. Renewable energy consumption, the pollution haven hypothesis, sustainable development, carbon dioxide emission, energy technology and environmental Kuznets curve are the current research frontiers in this field. In terms of the number of published papers, research in this field has been conducted in a national (regional) layout with China as the core force, and Italy, America, Britain, Germany and other European countries as important forces. This field covers three main research areas: enterprise performance, policy instruments and research methods, going through the start-up phase (2001–2011), the growth phase (2012–2018) and the development phase (2019–2021). Future research can further incorporate the digital economy and synergy of multiple environmental regulation policies into this field, which will continuously enrich the theoretical research system in this field. The content, methods and conclusions of research in this field are becoming increasingly diverse.

## 1. Introduction

At present, countries around the world have made remarkable achievements in economic construction. However, environmental degradation and excessive consumption of resources have become a new problem for the era, seriously restricting the sustainable development of society. For this reason, countries have followed the concept of green development, actively explored scientific environmental regulation systems suitable for healthy economic and social development and made a series of strategic plans. Various laws and regulations, environmental standards and local systems relating to environmental protection have emerged. International environmental policy is an important part of environmental protection policies. The Basel Convention on the Control of Transboundary Movements of Hazardous Wastes and Their Disposal (Basel Convention) of 1989 required countries to minimize the number of hazardous wastes, and to store and deal with it locally as much as possible in the most environmentally friendly way. The United Nations Framework Convention on Climate Change (UNFCCC), promulgated by the United Nations Conference on Environment and Development (UNCED) in 1992, set out the ultimate goal and fundamental principles for addressing climate change. In the same year, the United Nations Conference on Environment and Development adopted the Convention on Biological Diversity (CBD), an international convention to conserve the Earth’s biological resources to the maximum extent possible. Countries actively comply with the conventions and are committed to environmental protection and restoration.

Aiming at efficiency, harmony and sustainability, green development is a mode of economic growth and social development. Currently, green development has gradually become a significant trend. The concept of green development takes the harmony between man and nature as its value orientation, the green low-carbon cycle as its primary principle and the construction of ecological civilization as its basic principle. Only by vigorously developing a green economy can all sectors effectively make a breakthrough in terms of the bottleneck constraints on different resources and the environment, take the initiative and establish an advantageous position in long-term economic and social development. The formulation and implementation of environmental regulation policies is a game of chance between the relevant interest groups, which ultimately manifests itself in the different environmental regulation policy choices of each country. Regulatory policies that work well in developed countries are not necessarily applicable to developing countries. Countries need to choose and design reasonable regulatory policies according to their own political, economic and social conditions. The key to the implementation of environmental regulations lies in whether these regulatory policies can effectively promote green technological innovation in enterprises, which in turn will lead to a “win-win” situation in terms of environmental protection and technological upgrading, and thus promote the construction of an ecological civilization. Although environmental regulation policies restrict and regulate production by enterprises to varying degrees, the government has supported and incentivized enterprises’ technological innovation and green transformation, via measures such as the implementation of government subsidies and tax relief policies. Enterprises have incorporated environmental responsibility into the development strategy planning. In the short term, enterprises need a large number of scientific research personnel and funds for R&D. Undoubtedly, enterprises need to invest huge funds and even face financing difficulties. In the long run, the promotion of GTI contributes to the outstanding transformation and advancement of enterprises and the optimization of production modes. Its income comes not only in terms of economic profits, but also the recognition of the public and its long-term development. At present, the research theme of ER affecting GTI has yielded abundant results, but the findings are not consistent. In view of the large number of research results that have emerged, a systematic review and summary is necessary. Therefore, this paper takes 738 papers from 2001 to 2021 in the core database of Web of Science (SCI-EXPANDED, SSCI) as research samples. Based on the bibliometric software CiteSpace, this paper makes a visual analysis of institutions, authors, keywords, countries (regions), journals, the annual number of papers and other multi-dimensional perspectives to explore the research hotspots, development trends and overall characteristics of ER affecting GTI, and finally draws the corresponding research conclusions and future research directions.

This study is structured into seven sections. Section 1 gives a brief introduction to the research background. Section 2 outlines the literature. Section 3 covers the data sources and research method. Section 4 presents the annual number of papers, authors, institutions, national/regional distribution and journal distribution of the research field of the impact of ER on GTI based on the bibliometric software CiteSpace. Section 5 presents the research hotspots of the research field based on CiteSpace. Section 6 presents the evolution of research themes and trends of the field based on CiteSpace. Section 7 outlines the conclusions and recommendations of the study.

## 2. Literature Review

Green technology innovation aims at the green development of the whole of society, promoting resource conservation, energy conservation and emission reduction, reducing energy consumption in various industries, achieving social pollution prevention and enterprise technology upgrading, and reflecting the two attributes of being green and innovative (Wang, Wu, & Li, 2021) [1]. Green technological innovation follows the ecological principles and the development rules of ecological economy (Liu, Zhu, Yang, & Wang, 2022). Wang et al. defined green technological innovation in two dimensions, broad and narrow, and the broad perspective considered GTI as technological innovation behaviors that save resources and protect the environment [2]. The narrow perspective considered GTI as innovations related to environmental technologies, processes and products embodied in the whole process of producing products or providing services in enterprises.

According to the Porter hypothesis, the state and the government regulate and restrain the production and operation behavior of enterprises in order to promote green development, and ER is currently an important tool for environmental governance (Porter & Van der Linde, 1995) [3]. Academic research on the impact of ER on GTI has reached inconsistent conclusions. There are mainly four viewpoints.

Firstly, ER positively promotes GTI. “The Porter hypothesis” states that a well-designed ER policy can stimulate GTI, improve the competitiveness of enterprises in the market and offset the costs of environmental regulation, i.e., produce an innovation compensation effect [4]. Porter et al. also showed that environmental regulation did not increase business costs, but rather promoted technological innovation and gave firms a competitive advantage [5]. Yuan et al. argued that firms were compelled by environmental regulations to actively research and develop technologies to reduce pollution treatment costs and ultimately improve GTI, validating the Porter hypothesis [6].

In the short term, the implementation of ER policies would increase the cost of GTI and crowd out R&D investment. However, in the long term, the positive interaction between ER and green innovation activities would promote technological progress [7], i.e., the “innovation compensation” effect. In general, increasing environmental supervision could improve corporate performance by stimulating GTI [8]. The government had promoted active GTI in all industries through both pollution taxes and fees as well as innovation incentives [9]. Government’s energy-saving and low-carbon policies not only effectively promoted GTI in China’s eastern and central regions and large-scale enterprises, but also enhanced the efficiency of technological innovation in technology-intensive industries and mature enterprises [10].

The reform of environmental protection tax had been improving the efficiency of fossil energy use and end-treatment innovation of enterprises, and effectively promoted green innovation activities [11]. Barbieri proposed that higher tax-containing fuel prices could effectively shift patent activities from non-green technology to green technology [12]. Based on the research of Dutch enterprises, Leeuwen et al. pointed out that ER had a positive impact on the efficiency of GTI, promoted the ecological investment of enterprises and carried out innovation to reduce pollution and save resources [13]. Yang et al. found that the water ecological civilization city pilot policy significantly contributed to the growth of the number of green patent applications in the pilot cities, indicating that the policy could significantly improve the green innovation capacity of cities [14].

The impact of ER on GTI is heterogeneous, reflected in different types of environmental regulations, different kinds of green technology innovation, as well as regional differences and industry differences. In addition, there are significant differences in the findings of scholars’ studies. In the case of China’s manufacturing industry [15], Yuan et al. used panel data from 2003–2013 for 28 sub-sectors of China’s manufacturing industry to classify them into three groups of high-, medium- and low-eco-efficiency industries based on their eco-efficiency levels [16]. It was found that for industries in the medium-eco-efficiency group, stricter environmental regulations could not only encourage technological innovation and economic benefits in manufacturing, but also promote a shift in focus of technological innovation away from traditional technologies to energy-efficient and environmentally friendly technologies, and such technological innovation could optimize economic and environmental performance. The study for the Yangtze River Delta region [17] also yielded positive findings.

The stronger the resource advantage of enterprises, the more the pollution charge policy could force enterprises to create more green invention patents [18], and the green innovation effect was obvious. Bergek et al. suggested that well-designed environmental regulatory policies could induce firms to engage in environmentally friendly technological innovation, and significantly improved their ability to innovate in green technologies [19]. Different types of instruments promote different types of innovation: general economic instruments mainly encourage incremental innovation; general regulatory instruments mandate improvements on modular innovation; and technology-specific instruments appear to be required to support the development of entirely new technologies. Zhang et al. showed that the stronger the economic incentive-based regulation, the more it would positively promote green technology innovation [3].

Liang et al. classified GTI into green process innovation before production, green product during production, and end-of-production governance technological innovation according to the process of commodity generation [20]. In addition, command-and-control environmental regulations can greatly improve technological innovation before and after the production of a firm’s products, while market-incentive environmental regulations can promote technological innovation before, during and post-production technological innovation. Xiong et al. constructed a triple difference model based on the quasi-natural experiment of China’s low-carbon pilot city policy, and the policy significantly improved GTI level of high-carbon emission enterprises in the pilot city, which verified the “Porter Hypothesis” [21]. The heterogeneity analysis shows that the low-carbon pilot city policy significantly improves GTI of high-carbon emission enterprises in the eastern and western pilot cities (especially non-state-owned enterprises).

Secondly, ER negatively inhibits GTI. ER led to increased costs, reduced profits and even pushed firms to the brink of bankruptcy [22], reducing firms’ investment in innovation, i.e., the “cost of compliance” effect. Ramanathan et al. examined the link between regulation, innovation and performance in the UK, and in the short term, ER would have a negative impact on innovation, which in turn had a negative impact on the economic performance of these sectors [23]. It has also been found that environmental regulations severely hindered GTI in moderately polluting industries [15] and highly patent-intensive manufacturing [24]. Zhang et al. suggested that direct regulation was not conducive to green technological innovation by firms and that this type of environmental regulation was a weak disincentive for non-Middle and Lower Yangtze River regions in central and western China [3].

Thirdly, the impact of ER on GTI is uncertain. The relationship between ER and GTI is nonlinear. Gong et al. pointed out that the relationship was “U” type [25]. Whereas Wang et al. found an inverted U-shaped relationship between the two [26]. The interaction between ER and government R&D would promote green product innovation and inhibit green process innovation, which was closely related to the imbalance of ER intensity in energy conservation and emission reduction [27]. Cecere suggested that regulatory stringency had a positive impact on innovation, but that this impact was non-linear, suggesting that there was an optimal upper limit to regulatory stringency [28].

The impact of ER on GTI varied by region [29]. Specifically, in the central and central coastal regions of China, there was an inverted “U”-shaped relationship between ER and GTI. In contrast, the northern, southern coastal and southwestern regions of China showed a “U”-shaped relationship between the two, which was not significant in the Beijing-Tianjin region [26]. There was a threshold effect of direct regulation on green technology innovation of firms in western China [30]. The relationship between command-based regulation and GTI was inverted U-shaped, and the relationship between investment-based regulation and GTI was U-shaped [31]. Wang et al. found that there was a nonlinear relationship between ER and GTI in some enterprises, and it was constantly adjusted with time [2]. ER had a “U” type relationship with GTI (sulfur dioxide emission intensity, industrial wastewater emission intensity, industrial wastewater removal rate). ER had a “U” nonlinear effect on green process innovation, and based on government subsidies, ER had a threshold effect on green process innovation and so regulations and subsidies should be increased [32].

Fourthly, there is no obvious relationship between ER and GTI. Petroni et al. proposed that the validity of the Porter hypothesis could not be proved under any conditions [33]. Sun found that the impact of ER on technological innovation was almost zero [34]. Pan et al. pointed out that in high-pollution areas, the impact of ER on green innovation was not obvious [35]. Brunnermeier et al. argued that increasing monitoring and enforcement activities related to existing laws and regulations did not provide any additional incentive for innovation [36]. In addition, although the emission trading mechanism improved the allocation efficiency of sulfur dioxide emission rights, the pilot policy of sulfur dioxide emission trading had not produced Potter effect in China [37].

Other scholars had found that ER also had a moderating effect on GTI such as ER, which had a positive moderating effect on the spillover of foreign direct investment into light-pollution industries to promote GTI [15]. Moreover, there was an interactive relationship between ER and GTI. Technological innovation of enterprises would further strengthen environmental supervision [38].

In summary, the current academic research on the impact of ER on GTI is gradually increasing, and the research methods are mainly qualitative research and quantitative research. The formation of the relationship between ER and GTI can be influenced by a variety of factors such as the type and intensity of environmental regulation, the type of green technology innovation, industry heterogeneity, and regional heterogeneity. With the promulgation and implementation of relevant national policies, academic circles will conduct diversified research into their impact. CiteSpace has been applied to the study of environmental regulation [39], environmental transparency [40], green innovation [41], green technology [42], and the combination of environmental regulation and green innovation [43], but there is few such literature. Based on CiteSpace, Li et al. revealed the publication trend of a number of Chinese green technology research papers, the evolution path of hot research topics, and the number of Chinese scholars on green technology research showed a N-type upward trend and will continue to rise in the future [44]. In addition, scholars have applied Citespace to other fields such as “optimization of spatial pattern of land use” [45] and “vision and sport” [46].

The shortcomings of previous literature studies are mainly in three aspects. In the field of environmental regulation affecting green technology innovation, firstly, existing studies are more often conducted using panel data and lack of analysis of the dynamic evolution of hot spots. Secondly, the literature reviews mostly adopt qualitative research, and lack quantitative analysis and visualization analysis. Thirdly, the current papers in this field involving Citespace are scattered in terms of topics, such as environmental regulation, green innovation, and green technology, and fewer papers combine environmental regulation with green technology innovation. Based on the existing literature, firstly, this paper focuses on the study of environmental regulation affecting green technology innovation. It further investigates the subcategory of green innovation, which is a better representation of a firm’s ability to innovate under the constraints of government environmental regulation at the technological level. Secondly, this paper synthesizes many applications of Citespace to provide a detailed analysis of the topic. Thirdly, this paper provides further suggestions for future research directions and in-depth studies in this field.

Green technology, technological innovation and environmental protection have long been the research hotspots of green technology in China. The knowledge base of green technology research in China is mainly composed of efficiency, the relationship between environment and economy, green technology innovation, environmental regulation and intellectual property rights. However, there are a few literature studies on the characteristics and development context of environmental regulation affecting green technology innovation. Against the background that countries vigorously promote environmental protection and enterprises’ green technology innovation capability, the in-depth study of the development context and research situation in this field has become an important issue. Therefore, this paper collects the literature on the impact of ER on GTI in Web of Science and conducts bibliometric analysis of this field based on CiteSpace to show the hotspots, frontiers and development trends of this field.

## 3. Data Sources and Research Method

### 3.1. Data Source

The retrieved data in this paper are from the two core databases of Web of Science (https://www.webofscience.com/wos/alldb/basic-search (accessed on 28 March 2022), and the related literature in this database is mainly analyzed by metrology and statistics. Data acquisition and validation steps are as follows. Firstly, this paper determined the retrieval expression. The subject words were preliminarily retrieved, and the words related to the subject words were identified and then the second retrieval was conducted. The keywords related to green technology innovation are “green technology innovation” and “green patent”. The key words related to environmental regulation are “environmental regulation” and “environmental policy”. The initial retrieval formula is formed through the above keywords, and the final retrieval expression is TS = (environmental regulation or environmental policy) and (green technology innovation or green patent). The language, literature type, time span and retrieval results of this study are detailed in Table 1. Secondly, the retrieved samples were imported into CiteSpace and reprocessed. The results showed that there were no duplicate papers, which were used as the sample data for the visualization research in this field.

### 3.2. Research Method

Bibliometric analysis is a basic and effective method to detect and investigate the development of a research field. CiteSpace is a citation visualization analysis software developed under the background of scientometrics, data and information visualization, focusing on analyzing the potential knowledge contained in scientific analysis [47]. The obtained visualization graph is called a “scientific knowledge map”. CiteSpace can show the development trend of a discipline or field in a certain period of time, analyze the development context of the discipline and identify the knowledge base, research hotspots and research frontiers of the discipline. The analysis software can find out the relationship between information in the literature, and present the relationship by using the map, which can be used to reflect the objective situation of scientific development. Citespace is designed to allow users to see how the knowledge graph will change the way they see the world by mapping, generating and interpreting knowledge graphs. The core function of CiteSpace is to provide a visual map of the evolution of a knowledge domain, with a higher level of abstraction of the “second-order science” category and more vivid visual images, to reflect and approach the scientific development of a specific area of the physical world in a deeper way [47,48,49]. The colors in the map can distinguish different nodes, connections, clusters, and also provide an aesthetic effect.

Based on the bibliometric software CiteSpace, this paper makes a visual analysis of 738 papers in the core database of Web of Science (SCI-EXPANDED, SSCI) in the field of ER affecting GTI. This paper mainly analyses the overview analysis of the field (the annual number of papers, national/regional distribution, co-cited journals analysis, co-cited authors analysis, institutional cooperation analysis, keyword co-occurrence analysis, keyword clustering analysis, thematic chronological evolution and other dimensions) to explore the research hotspots, development trends and overall characteristics of ER affecting GTI. The time interval was set from 1 January 2001 to 31 December 2021, and the time slice was 1 year.

## 4. Analysis of the Impact of Environmental Regulation on Green Technology Innovation

### 4.1. Analysis of the Annual Number of Publications

**Figure 1:**
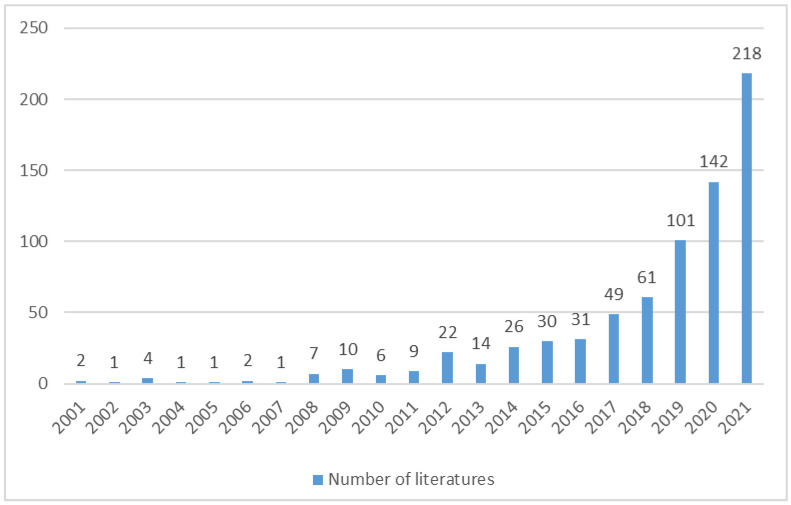
The number of published papers from 2001 to 2021.

The changes in the number of academic literatures in a research field to some extent illustrate the stage, trend and development speed of academic research in this field from a macro perspective. The analysis of the number of papers reveals the development trend and attention in this field. Figure 1 reflects the overall research trend of Environmental Regulation affecting Green Technology Innovation in the 21 years. As can be seen from Figure 1, there are three main phases of research in this research area. The first stage was from 2001 to 2011, during which the number of publications was not significant, and the average annual number of publications was low, with less than five papers published in most years. This indicates that the research team in the field of ER influencing GTI research was small in the early stage, and the academic community had not yet entered a research boom as there were few studies in this field. The second stage was 2012–2018, with an average annual number of 33 papers, which was greatly improved compared with the first stage. The third phase, since 2019, had averaged over 100 papers per year. The highest number of publications was 218 in 2021. This field has become a research hotspot. Overall, the number of publications (research findings) in this area is increasing.

### 4.2. Analysis of the Authors of Literature

**Figure 2:**
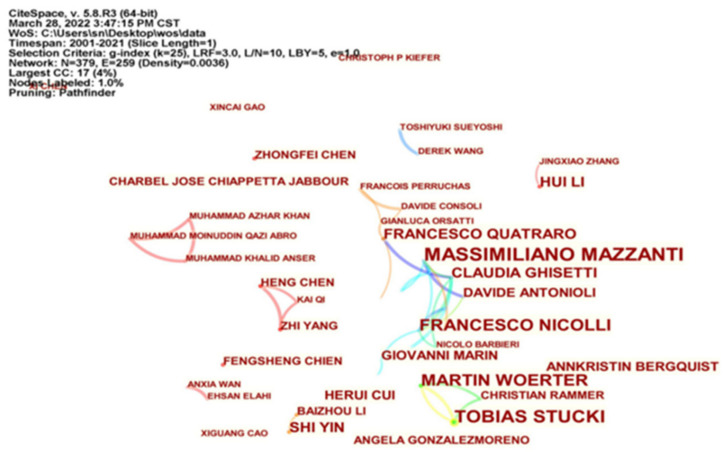
CiteSpace-based author collaboration mapping.

**Figure 3:**
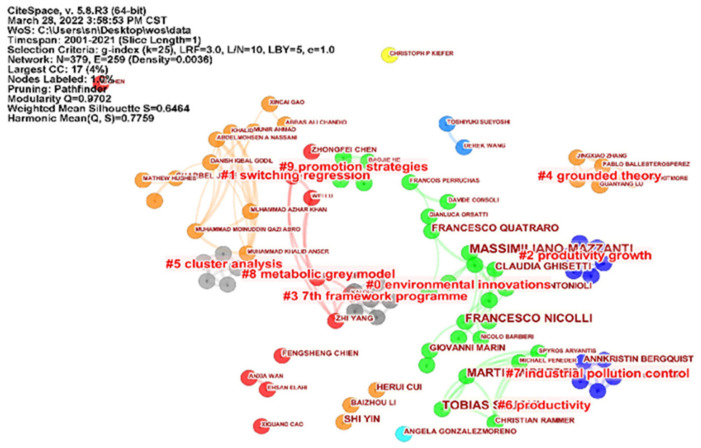
CiteSpace-based author collaboration clustering mapping.

Firstly, the authors’ cooperation visualization map (Figure 2) and authors’ cooperation clustering view (Figure 3) of the impact of environmental regulation on green technological innovation are drawn by CiteSpace. 5.8.R3. The author cooperation network reflects the cooperation of scholars in the field of a discipline, and the number of papers reflects the research input of scholars in this field.

On the one hand, Table 2 reflects the number of individual authors’ published papers. The top four scholars are Massimiliano Mazzanti, Tobias Stucki, Francesco Nicolli and Martin Woerter. The number of published papers is 7 or more, and the highest number is 10. Most scholars only publish one paper. Among these scholars, Mazzanti et al. found that environmental policies adopted by countries could contribute directly and indirectly to the performance of environmental productivity through innovation [50]. Over the last decades, environmental policies have become increasingly stringent and have been an important driver of climate action, waste and resource efficiency changes, in relation to various sustainable development goals about consumption and production, economic growth, industrial and infrastructural innovation and climate action. Based on the data of representative companies in Austria, Germany and Switzerland, Stucki et al. found that policies may affect green product innovation by directly stimulating the supply of green products or services, or more indirectly by stimulating the demand for green products or services [51]. After controlling the demand-side effect, taxes and regulations were negatively correlated with green product innovation. Woerter et al. found that an energy tax was a very effective policy tool to promote the diffusion of green energy technology within the company through the specialized survey data of 1200 Swiss companies [52].

On the other hand, as far as author cooperation is concerned, in Figure 2, the node size is positively correlated with the number of papers published by authors in a subject field, the node connection represents the cooperative relationship between authors, and the thickness of the connection is positively correlated with the intensity of author cooperation. In the research field of ER affecting GTI, the number of nodes is 379, the node connection is 259, and the network density is 0.0036. It can be seen from Figure 2 that the number of nodes and connections is large, but the network density is low, and the network structure is relatively dispersed. It shows that although many scholars in this field have studied and cooperated to some extent, the cooperation relationship is not close.

On the basis of Figure 2, the author’s cooperative clustering view of Figure 3 is further formed. There are 10 clustering blocks in Figure 3. Cluster #0-Cluster #9 represent cooperative groups in different research directions, and the groups are also relatively large. The groups in this area are generally small, with most of the authors publishing independently. The largest group is a 17-author group with a focus on environmental innovations, followed by a 12-author group with a focus on switching regression, and a six-author group with two research interests: productivity growth and the 7th framework programme. The other groups are working on grounded theory, cluster analysis, industrial pollution control, promotion strategies, metabolic grey model and productivity. In summary, a number of small-scale collaborative networks have formed in the research field of ER influencing GTI. There are relatively few larger collaborative teams, with a predominance of two-person and three-person research groups, but most scholars affiliated with institutions appear as independent authors, and most authors have a relatively low frequency of publications.

**Figure 4:**
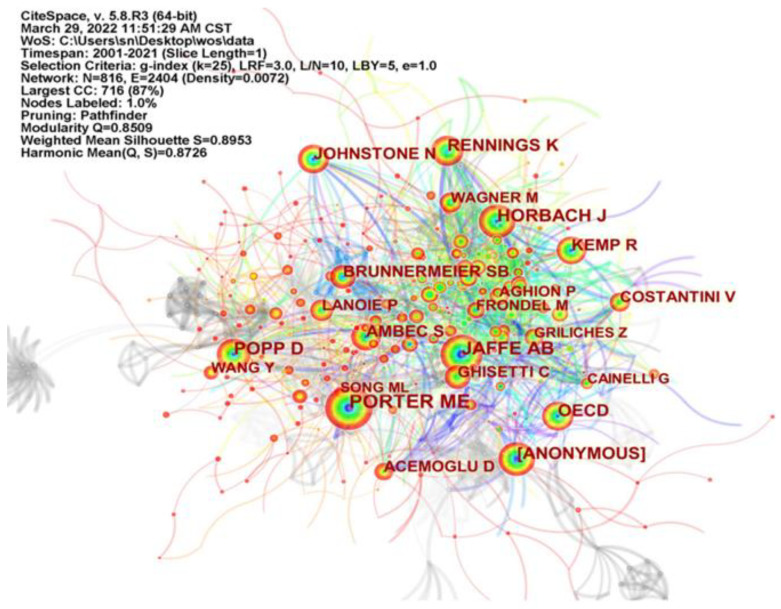
CiteSpace-based author co-citation mapping.

Secondly, Figure 4 further generates an author co-citation map of research on environmental regulation affecting green technology innovation through CiteSpace software. The size of the nodes in the graph represents the number of times an author has been cited. The larger the nodes, the higher the number of citations. Table 3 shows the frequency and centrality of TOP 12 co-cited authors, with higher centrality (above 0.1) being more important. Combining Figure 4 and Table 3, it can be seen that there are nine scholars with a total of 100 citations and above. The most notable is Porter ME with a total citation frequency of 277 and a high centrality (0.12). The second most cited author is Jaffe AB with 277 total citations. In addition, scholars with high co-citation frequency include Horbach J, Popp D, and Rennings K. Among these scholars, Popp et al. used renewable energy as an example and found that different types of policy instruments were effective for different renewable energy sources [53]. Broad policies, such as tradable energy certificates, were more likely to induce innovation in technologies that come close to competing with fossil fuels. Therefore, more targeted subsidies, such as feed-in tariffs, were needed to induce innovation in more expensive energy technologies, such as solar power. Ambec examined the main theoretical foundations and empirical evidence regarding Porter’s hypothesis, discussed its implications for the design of environmental regulations, and outlined directions for future research on the relationship between environmental regulations, innovation and competitiveness [54]. Many achievements of these scholars provide the basis for the subsequent research on the impact of ER on GTI.

### 4.3. Analysis of Literature Publishing Institutions

**Figure 5:**
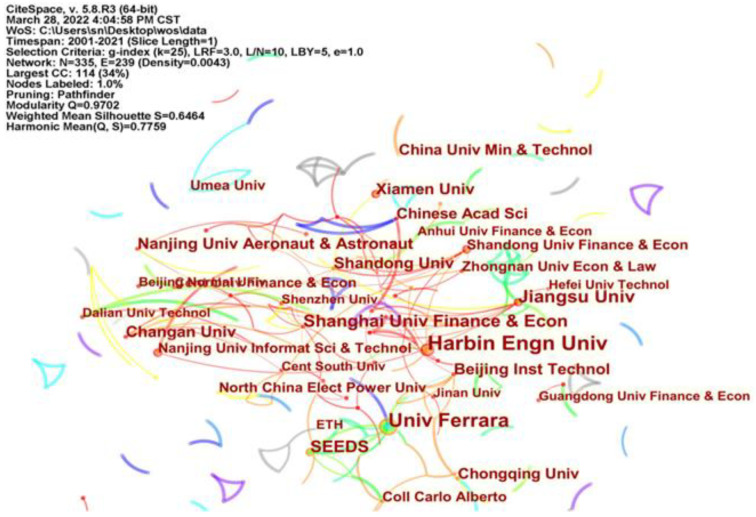
CiteSpace-based mapping of institutional collaboration.

The visual analysis of research institutions reveals the practical results and cooperation of many institutions within a discipline. First of all, from the perspective of cooperation density of research institutions, Figure 5 is the visualization map of institutional cooperation of the research into ER affecting GTI. In the figure, the nodes represent the research institutions, the number of nodes is 335, the node connection is 239, and the network density is 0.0043. The size of the nodes in the map reflects the number of papers published by each institution. The larger the node, the greater the number of papers published by the institution. The lines between nodes represent the collaboration between institutions. The strength of the lines is proportional to the strength of the collaboration. The number of nodes and the number of connecting lines in Figure 5 are high, but the network density is low, and the distribution of mechanisms is scattered. Many of the institutions in the figure do not cooperate closely with each other, and many of them have only one line of connection. It is clear that cooperation between institutions in the field of research on ER affecting GTI is very sparse. Secondly, the data on the volume of papers published by each institution are analysed. Table 4 lists the top ten research institutions in terms of the number of publications in this field, and the nodes of these ten institutions are larger. At the top of the list is Harbin Engineering University with 18 publications, followed by University of Ferrara with 15 publications and again by Jiangsu University with 12 publications. The top ten research institutions are predominantly Chinese institutions, with universities being the mainstay of research in this area. At the same time, the institutions in Table 4 collaborate closely with other institutions. For example, Harbin Engineering University collaborates with universities such as Shanghai University of Finance and Economics and Hebei Agricultural University. The University of Ferrara jointly conducts its study with the European Commission, OFCE SCi Po and other institutions. Jiangsu University cooperates with Data Link List, City University and Nanjing University of Information Science & Technology. Most organizations published less than 5 papers. Many research institutions only published one paper. Only a few institutions have a relatively high volume of papers. Academics are paying more attention to the study of ER affecting GTI and have ventured into this area of research.

### 4.4. Distribution of Countries (Regions) with Research Findings

**Figure 6:**
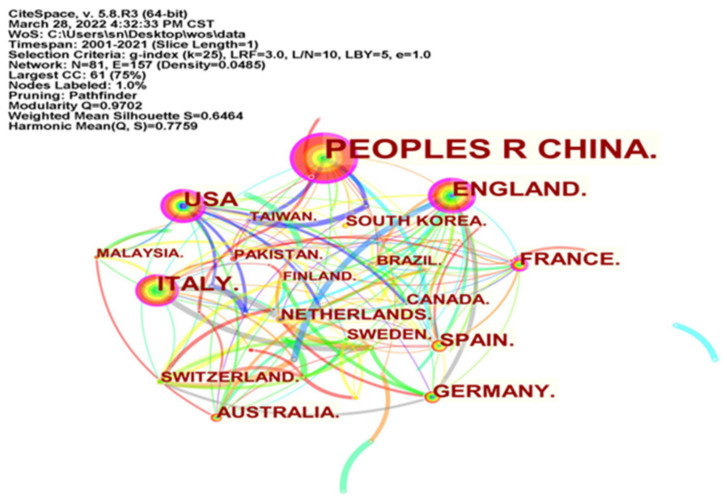
CiteSpace-based country (regional) cooperation mapping.

The national (regional) distribution of research results can reflect the degree of development of the field in a specific country (region). The national (regional) distribution of research in the field of green technology innovation influenced by environmental regulation is obtained from CiteSpace software, as shown in Figure 6. The node size reflects the number of papers published in the country (region). The line between nodes indicates the cooperation between countries (regions). The thickness of the linkage indicates the strength of the cooperation relationship. The centrality indicates the importance of a country in the map. Table 5 shows the top 12 countries (regions) in terms of the number of papers published. Combining the results in Figure 6 and Table 5, firstly, it can be seen that the literature on ER affecting GTI is mainly from China, with 301 papers published in China, nearly one-half of the research sample. Secondly, European and American countries such as Italy, the United States and the United Kingdom have published a relatively large number of papers, 72, 71 and 68 respectively. Other European countries (Germany, Spain, France, Switzerland, The Netherlands, etc.) have scattered publications, but the overall research is relatively rich. Asian countries such as South Korea have 17 papers. The centrality of China (0.25), Italy (0.15), the United States (0.24), the United Kingdom (0.29) and France (0.16) are higher than 0.1, indicating that the research results of these five countries are more prominent in the field of ER affecting GTI. In addition, there is active cooperation between countries; for example, scholars from France and Spain have cooperated in research in this field. In summary, in terms of the number of published papers, research in the field of ER affecting GTI has formed a national (regional) layout with China as the core force and Italy, the United States, the United Kingdom, Germany and other European and American countries as important forces.

### 4.5. Journal Distribution of Available Research

**Figure 7:**
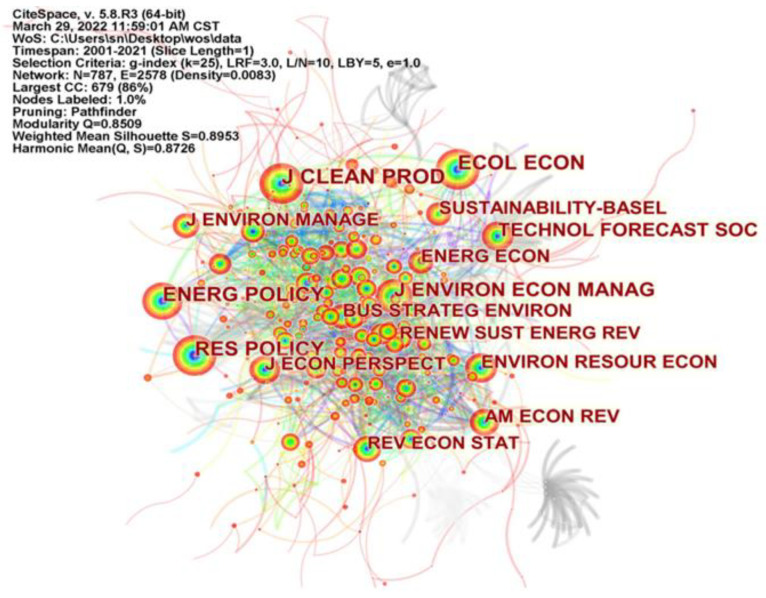
CiteSpace-based journal co-citation mapping.

Figure 7 generates a map of co-cited journals for research on environmental regulation affecting green technology innovation using CiteSpace software. The journals shown in the mapping are abbreviations of the journal names. Nodes represent the cited journals. The higher the number of citations, the larger the nodes. It is clear that there are many highly cited journals in the field of ER influencing GTI research. Table 6 shows the co-citation frequency, centrality and impact factor of the top 10 journals on the topic of ER affecting GTI, with the full names of the journals shown in the table. A total of 33 journals were cited at a frequency of 100 and above. Journal of Cleaner Production is one of the top journals in environmental studies as it is the most cited journal with a total of 489 citations and a high centrality (0.11) between 2001–2021. The journal is an international, interdisciplinary journal focusing on research and practice in clean production, environment and sustainability. It aims to encourage innovation and creativity, new and improved products, and the implementation of new and cleaner structures, systems, processes, products and services. The second most co-cited journal is Ecological Economics, a top journal in the field of ecological economics with 398 co-citations, which emphasizes key work drawing on and integrating elements of ecological science, economics and the analysis of values, behavior, cultural practices, institutional structures and social dynamics. In addition, other top international journals in the environmental category such as Energy Policy, Environmental & Resource Economics and Journal of Environmental Economics and Management have a total citation frequency of 381, 236 and 302 respectively. The top economic journals such as American Economics Review and Journal of Economic Perspectives are cited at 233 and 230 respectively, with the former having a centrality of 0.17. It shows that the field of research on ER influencing GTI is an integrated and cross-cutting field that integrates multiple theories from ecological studies, management science and business studies. At the same time, these highly cited journals have higher impact factors, indicating that these journals are high-quality journals in various disciplines, with greater influence and more credible research results. In summary, the above journals constitute an important carrier of the research into ER affecting GTI.

## 5. Analysis of Research Hotspots

### 5.1. Keyword Co-Occurrence Analysis

**Figure 8:**
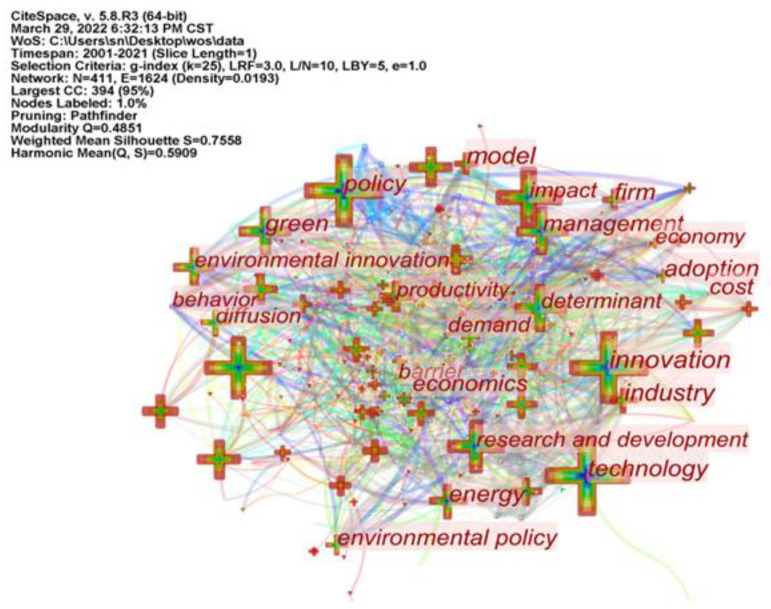
CiteSpace-based keyword co-occurrence mapping.

Keywords are a high-level summary and distillation of research topics in a specific research field, revealing the research hotspots and trends in the subject area. The keywords were imported into CiteSpace software with the following parameters: Node Types as Keyword, Timeslicing as January 2001–December 2021, and Time Slice as 1 year, to generate a keyword co-occurrence knowledge map (Figure 8) and to collate a high-frequency keyword ranking table (Table 7). The size of the nodes in the graph represents the frequency of the keyword appearances. The higher the frequency, the larger the node circle. Keywords with a high frequency are hot spots for research in the field. The lines between nodes indicate the co-occurrence of keywords. The number and thickness of the lines between the nodes reflect the strength of association between the keywords. The number of nodes in Figure 8 is 411, and the number of connected lines is 1624. The more the network density in the knowledge graph takes a value close to 1, the stronger the tightness between nodes. The network density in the graph is 0.0193, which is relatively low, indicating that the keywords are not closely connected to each other. In the graph, “innovation” and “impact” are the largest nodes, followed by “technology”, “performance”, etc. These keywords are of high interest in the field and are the focus of research. Combining the frequency of keywords (Table 7), the keywords innovation (165 times), impact (165 times), technology (149 times), performance (141 times), policy (139 times) and environmental regulation (66 times) have a high frequency and are important nodes in the network, as well as being research hotspots in the field of ER affecting GTI research.

The network centrality indicator in the knowledge graph reflects the importance of each keyword in the network structure. In general, keywords with an indicator (Centrality) value above 0.1 are more influential. As can be seen from Table 7, the centrality values of very few keywords such as innovation (0.15) and energy (0.11) are higher than 0.1, which also indicates that the current research is mainly focused on the government’s support of enterprises’ green technological innovation in the energy industry and even in other industries through multiple policies such as penalties and incentives to promote energy efficiency in various industries.

### 5.2. Cluster Analysis of Keywords

**Figure 9:**
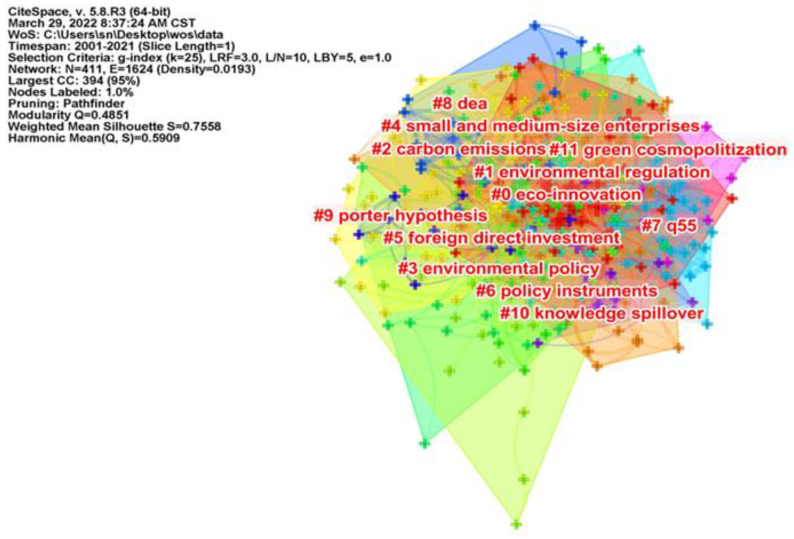
CiteSpace-based keyword clustering mapping.

Based on the analysis of keyword co-occurrence analysis, in order to show the connection between keywords more intuitively, the keyword cluster analysis mapping was obtained by CiteSpace software, which can understand the research hotspots in the field of ER affecting GTI research from a more macro perspective. Cluster labels are named after the keywords with the higher arithmetic value. The smaller the number of cluster labels, the more keywords are included in the cluster. Each cluster in the atlas consists of a series of strongly related keywords, and the different clusters represent different research directions within a subject area. Cluster labels are named after the keyword with the larger arithmetic value. In the map, each cluster is composed of a series of keywords with strong correlation. Citespace uses Modularity (Q value) and Silhouette (SM value) to judge the map rendering effect. It is generally believed that Q value > 0.3 means that the clustering structure is obvious, S value > 0.5 means that clustering is reasonable, and S > 0.7 means that clustering is convincing. It can be seen from the clustering view in Figure 9 that Q = 0.4851, S = 0.7558, indicating that the clustering of ER affecting GTI research is obvious and convincing.

There are 12 clusters in the map, namely eco-innovation, environmental regulation, carbon emissions, environmental policy, small and medium-sized enterprises, foreign direct investment, policy instruments, q55, dea, porter hypothesis, knowledge spillover and green cosmopolitization, which are the hot research areas in the international academic field of ER affecting GTI.

### 5.3. Timeline Mapping Analysis

**Figure 10:**
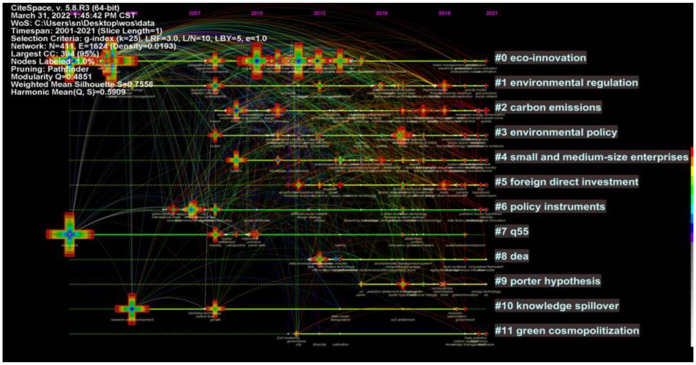
CiteSpace-based timeline mapping.

Using Citespace software, a timeline mapping of the research area of environmental regulation affecting green technology innovation was formed on the basis of Figure 9, resulting in Figure 10. The right-hand side of Figure 10 shows twelve clusters, representing the different research directions formed within the area. As can be seen from the keywords in the chart, currently, based on previous research, renewable energy consumption, the pollution haven hypothesis, sustainable development, CO_2_ emission, energy technology, the environmental Kuznets curve, and total factor productivity constitute the research frontiers in this field for the period 2018–2021. By combing through the retrieved 738 papers and combining the results with the high-frequency keywords ranking table, the keywords in Figure 10 are divided into the following three research categories: enterprise performance, policy instruments and research methods.

(1) Enterprise performance mainly involves clustering “#0 eco-innovation”, “#4 small and medium-size enterprises”, “#5 foreign direct investment”, “#7q55”, “#10 knowledge spillover”, including keywords such as competitive advantage, foreign direct investment, backstop technology, research and development. In the context of China’s launch of the Green Technology Bank (GTB) in 2016, which established an online database of green technologies, Guo et al. designed a three-tier classification system for green technologies (CSGT) in order to provide standardized classification criteria for these technologies, which classified green technologies into five main categories, including environmental quality, resource use, energy use, life health and ecological safety [55]. It can be seen that CSGT brought multiple performance of enterprises and society. At present, countries around the world attach importance to science and technology innovation as the driving force to promote the transformation and upgrading of energy resources, industrial structures and consumption structures, to promote green economic and social development, and to explore a new path of synergy between development and protection. Green technology innovation will bring “double benefits” in terms of technological progress and environmental protection, thus breaking through the “economic-environmental” pressure and achieving the synergistic development of the economy and ecology. The importance that enterprises attach to R&D innovation is not only in line with the inevitable trend of upgrading the economic structure, but also for the enterprises themselves to find opportunities and breakthroughs to survive in the fierce market competition. Although the initial investment may be costly, the value brought by R&D results can enable enterprises to have a differentiated core competitiveness. At the same time, the results of innovation can align the internal and external resources of a company to achieve optimal allocation. New technologies can also reduce future operating costs and bring more benefits to the company. Improving R&D investment must improve the utilization of R&D resources so that R&D results can be turned into commercial value more quickly. On this basis, enterprises can also attract more external capital to join them in technological innovation. Green technology innovation can not only improve R&D performance and thus bring significant financial performance, but also generate environmental performance, provide abundant green products and services to society and actively promote green concepts.

(2) Policy instruments mainly involves clustering “#1 environmental regulation”, “#2 carbon emissions”, “#3 environmental policy”, “#6 policy instruments”, and “#11 green cosmopolitization”, including keywords such as environmental management, renewable energy consumption, green strategy, haze pollution, and city diversity. In accordance with national conditions, countries around the world have widely adopted various environmental policies to prevent and control pollution, using economic and administrative instruments (such as environmental taxes and fines) to require pollutant emitters to bear responsibility for the social damage caused by pollution. These practices aim to encourage polluters to reduce environmental pollution from production by optimizing technological structures, energy structures and product structures, and to achieve the internalization of the external diseconomies of environmental costs. Drawing on Israeli standards on environmental pollutants in consumer goods and “green buildings”, Goulden et al. considered how the use of standards as an environmental policy tool affected environmental regulation [56]. Runhaar et al. referred to environmental policy integration (EPI) as the integration of environmental issues into non-environmental policy sectors, aiming to avoid conflicts between environmental and other policy objectives and to strengthen environmental policy by directly targeting the drivers of environmental degradation [57]. In order to reduce environmental pollution, high-energy enterprises must improve their energy efficiency. Amowine et al. [58] and Li et al. [59] used DEA model to measure energy efficiency in African countries. Additionally, the government also provides R&D subsidies for companies in various industries to stimulate green technology innovation, achieve energy saving and reduce energy consumption. Also, Li et al. found that the influence of local government green development behavior was stronger than that of central government behavior [60]. 

(3) The research methodology contains mainly “#8 dea” and “#9 porter hypothesis”, with keywords such as environmental management system, porter hypothesis, panel, dea, etc. The DEA model has been widely used to measure the efficiency of green technology innovation, and the DEA model itself has been further developed. Since the introduction of the “Porter Hypothesis”, many scholars have studied it based on their own or other countries’ realities, with varying conclusions. Academic research methods in this area are becoming increasingly rich and are not limited to the traditional multiple regression and ordinary least-squares methods. Methods such as triple difference, simulation, evolutionary games, meta-analysis, and panel threshold models are also gradually being adopted by scholars. Deng at el. analyzed firms’ optimal green technology innovation strategies using Stackelberg game theory and optimization theory and found that controlling the relative size of local governments’ investment incentive coefficients in environmental governance and economic development could promote the positive impact of political competition on firms’ optimal level of green technology innovation [61]. Using a meta-analytic approach, Ghisetti found that regulatory stringency in particular affected environmental innovation. Research on the impact of ER on GTI has been increasingly rich [62].

## 6. Evolution of Research Themes and Trends

### 6.1. Thematic Chronological Evolution

**Figure 11:**
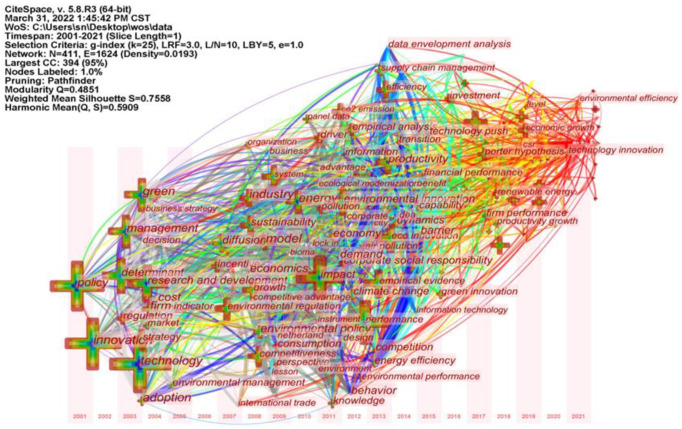
CiteSpace-based timezone mapping.

The keyword timezone mapping reveals the development history and thematic evolution of the environmental regulation influencing green technology innovation research field in each time period, and the timezone mapping is generated based on running CiteSpace for keyword clustering view, i.e., Figure 11. The mapping shows the dynamic process of the thematic evolution of how the ER influenced GTI research field and the layout characteristics of the keywords. The position of the node of a keyword in the knowledge graph is the time when the keyword first appeared in the research literature. The connecting line represents that the two keywords appear in the same paper. High-frequency keywords appear more often, and their location nodes are larger, while these keywords are also hotspots for research. The keyword timezone mapping in Figure 11 provides a more detailed picture of the evolutionary trend of research in the field, which can be divided into three main phases: the start-up phase (2001–2011), the growth phase (2012–2018), and the development phase (2019–2021). This coincides with the phased nature of the number of papers published in Figure 1.

Firstly, there is the start-up phase (2001–2011). There are few research results at this stage, as they mainly focus on environmental policies and enterprise R&D innovation, including representative keywords of green, pollution, environmental management, environmental policy, CO_2_ emission, environmental regulation, innovation, technology, research and development, and account for 6% of the total number of papers published. The research at this stage is generally in the initial exploratory stage. Countries around the world have introduced environmental regulation policies and are exploring in practice policies that are suitable for their national conditions. Kivimaa et al. noted that environmental policy integration in Finnish technology policy was empirically assessed by focusing on all levels of technology R&D support, from policy strategy to project funding decisions, but this integration was not comprehensive and environmental impact assessments were not required in funding applications [63]. Engel-Cox et al. proposed a science-policy data model that defined conditions for promoting the use of environmental monitoring data for policy, which could help scientists and policy makers diagnose barriers between science and policy and work together more effectively to use monitoring data for environmental policy [64]. At the same time, industry is the most significant area of energy consumption and greenhouse gas emissions such as carbon dioxide, and also bears a greater responsibility for the environment. Companies are also becoming aware of the importance of developing and upgrading green technology innovations.

Secondly, there is the growth phase (2012–2018). The research results at this stage have steadily increased, mainly focusing on corporate performance, empirical research and other topics, including firm performance, financial performance, investment, technology push, empirical analysis, data envelopment analysis and other representative keywords. The number of publications accounts for 32% of the total. Environmental regulation greatly affects the production and operation activities of enterprises, which in turn affects their business performance, financial performance and R&D performance. Based on this, academics have studied the relationship between environmental regulation and firm performance, exploring potential mechanisms and pathways to improve firm performance. Joo et al. found that eco-innovation was the strongest motivator for accelerating firm performance and that Chinese government support for firms appeared to be more influential than that of the Korean government [65]. Research methods include empirical studies such as least squares and panel threshold models, as well as data envelopment analysis to measure firm performance, such as the efficiency of green technological innovation and green total factor productivity. For example, Zhang et al. used the DEA-Malmquist method to measure the green total factor productivity of various food industries in China from 2006 to 2014, and the results showed that the environmental pollution index of the Chinese food industry showed an upward decreasing trend from 2006 to 2014 [66].

Thirdly, there is the development phase (2019–2021). This stage is in the high-yield period, and the research results are increasing rapidly. The research content shows multi-level and multi-angle characteristics, and integrates the results of the first two stages. This stage mainly focuses on topics such as environmental performance and scientific and technological innovation, including representative keywords such as environmental efficiency and technological innovation, accounting for 62% of the total number of papers published. Ong et al. aimed to investigate the relationship between firms’ environmental performance, environmental innovation and financial performance and found that environmental competitiveness, i.e., environmental innovation and environmental performance, are key factors in creating economic value for environmentally active manufacturing firms [67]. Xue et al. examined the impact of multidimensional corporate environmental performance (CEP) on firm risk using an observational sample of 1632 firms in the UK from 2002 to 2013, considering two dimensions of corporate environmental performance, namely environmental management performance (EMP) and environmental operational performance (EOP), and found that EMP is an effective mechanism for reducing firm risk and that this impact is mainly driven by the manufacturing sector [68]. At the same time, there was no significant association between EOP and business risk. Usman et al. pointed out that clean energy technology, GDP growth and globalization played an active role in increasing clean energy consumption [69]. Zhang et al. found that solar technology, carbon intensity of energy structure and economic expansion had long-term effects on carbon emissions [70]. In addition, environmental innovation was also found to mediate the translation of the benefits of environmental performance into financial performance. With the implementation of environmental regulation policies and the transformation of the results of GTI by enterprises, they have gained both environmental and economic effects and achieved technological breakthroughs, such as a significant increase in the number of green invention patents.

### 6.2. Analysis of Burst Words

**Figure 12:**
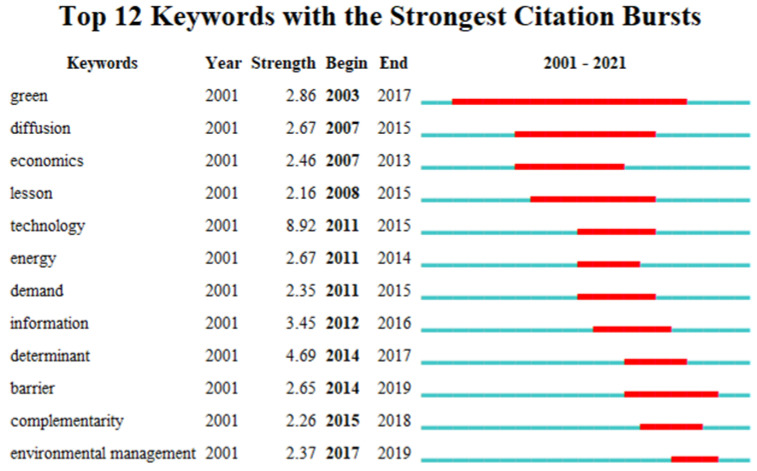
CiteSpace-based burst word mapping.

The analysis of burst words in a discipline field is based on the sudden outbreak of hot spots in this field, and the burst words can show the phased frontier research field of environmental regulation affecting green technological innovation. Using burst terms, 12 prominent words were detected in the keywords of the literature of ER affecting GTI (Figure 12), followed by green, diffusion, economics, lessons, technology, energy, demand, information, determinant, barrier, complementarity, and environmental management. The keywords in the map represent the research frontier in different periods. The red part represents the burst period of keywords, including the beginning, end and lasting period of keywords. The blue color represents no occurrence of burst. The “Year” in the map represents the time when the keywords first appear. “Begin” and “End” represent the year of onset and end. “Strength” represents burst strength. It can be seen from the map that “green” has emerged for the longest time (15 years), indicating that green development has become an important trend. Many countries regard the development of green industries as an important measure to promote economic restructuring. At the same time, the concept of green development has penetrated into all walks of life, promoting the green technology innovation of enterprises to assume environmental responsibility. Ospanova et al. proposed a component model of sustainable development that could reflect the essence and content of green economy, which summarized the positive experiences of green integration [71]. Lam et al. aimed to investigate the green marketing status of major ports in the world and suggested that ports should link the three basic aspects of strategy, structure and function in green marketing [72]. Promoting green development requires the active participation of countries around the world. Prominent words such as energy, diffusion, information and environmental management illustrate the practice of environmental regulation in countries around the world. For instance, the EU’s 2030 Climate and Energy Policy and the 2030 Agenda for Sustainable Development emphasized mitigation of climate change and reduction of its impacts by supporting the sustainable use of resources [73].

The regulation of environmentally sensitive industries such as paper-making and chemical industry is the focus of government environmental regulation. The government has continuously strengthened its supervision over enterprises. Enterprises need to disclose environmental information according to law, understand their environmental behavior and its impact in order to improve internal management, enhance the image of enterprises, and achieve the best combination of economic and environmental benefits. Figure 12 shows that the emergence intensity of technology is the highest (8.92). Many scholars have studied green technology. For instance, Wang et al. argued that green technology innovation of enterprises was very essential to promote enterprise competitiveness and environmental protection [74]. It can be seen that GTI can not only reduce costs, such as operating costs, environmental costs, resource utilization costs, but also improve market share, expand enterprise scale and achieve sustainable development. In addition, enterprises can transfer their green technology to obtain technology transfer benefits so as to promote the sustainable development of enterprises.

## 7. Conclusions and Recommendations

### 7.1. Conclusions

Based on the CiteSpace software, this paper conducts a visual analysis of 738 papers in the field of ER affecting GTI research from 2001 to 2021, analyzing the number of papers published in the field, authors, institutions as well as keywords, countries (regions) and journals, in order to study in depth the current situation, evolutionary lineage and development trends of this research field. The following main conclusions were drawn.

Firstly, in terms of the number of publications, the amount of literature in the field of ER influencing GTI research has shown an incremental trend and rapid growth. According to the search method of this paper, the two core databases of Web of Science (SCI and SSCI) in this field comprise 738 papers during 2001–2021. The number of papers published in the early period is low, and the total number of papers published in the three years 2019–2021 is 461. Currently, the field has entered a period of a research boom, the research momentum in the field is outstanding, and the number of papers published has increased significantly.

Secondly, in terms of authors and institutions, the core authors in this field are Massimiliano Mazzanti, Tobias Stucki, Francesco Nicolli and Martin Woerter. Although there are many collaborative groups working in different directions, such as environmental innovations, switching regression, productivity growth, the groups are relatively small, with a predominance of two-person and three-person research groups. Collaboration among scholars is weak, with most authors affiliated with institutions publishing independently. In addition, scholars such as Porter ME, Jaffe AB and Horbach J are highly cited authors whose research has laid the foundation for the field. Institutions with relatively high numbers of publications include Harbin Engineering University, University of Ferrara and Jiangsu University. The top ten research institutions are predominantly Chinese institutions, with universities being the mainstay of research in the field. Numerous research institutions have published papers alone.

Thirdly, in terms of national (regional) distribution and publishing journals, according to the number of published papers, research in the field of ER affecting GTI has formed a national (regional) layout with China as the core force, and Italy, the USA, the UK, Germany and other European and American countries as important forces. This field is a comprehensive and cross-cutting field that integrates ecological research, management science and business research and other multiple theories. The Journal of Cleaner Production, Ecological Economics, American Economics Review and other top international journals together constitute an important vehicle for research on ER influencing GTI.

Fourthly, from the perspective of research hotspots and development trends, the keywords of innovation, impact, technology, performance, policy and environmental regulation are the research hotspots in the field of ER affecting GTI. Research directions include eco-innovation, environmental regulation, carbon emission and environmental policy and other directions. This field has experienced a process from preliminary exploration to rapid development. The research can be divided into three phases: the start-up phase (2001–2011), the growth phase (2012–2018), and the development phase (2019–2021).

Fifthly, in terms of research content and research frontiers, the clustering of keywords for integrated analysis shows that research in the field of ER affecting GTI mainly involves three research categories: enterprise performance, policy instruments and research methods, and these three categories of research categories appear throughout the research. Renewable energy consumption, pollution hypothesis, sustainable development, CO_2_ emission, energy technology, and environmental Kuznets curve are the current research frontiers in this field.

### 7.2. Recommendations

Firstly, in terms of network density, the inter-author and inter-institutional network density in the field of ER affecting GTI research is low. The distribution of authors and institutions is relatively dispersed, which is consistent with the results of the network density analysis by Wang et al. [75]. Therefore, the current state of cooperation in this area should be stabilized and optimized. It should also focus on cultivating core teams and core authors, improving the knowledge creation ability of all authors, and enhancing the centrality and influence of each author. There should also be close communication between high-productivity groups and low-productivity groups to reduce the imbalance of research levels among research groups.

Secondly, the research results in the field of ER affecting GTI mainly focus on a few countries (regions) and scientific research groups. In the context of carbon neutrality and carbon peaking, countries can conduct research in this area based on their own situation, and also actively cooperate with other countries (regions), which can help to expand the international cooperation network. In particular, international transnational collaborations, interdisciplinary themes, and multi-author collaborations should be considered in areas such as environmental studies and ecology, which is consistent with the viewpoint of Jia et al. [76].

Thirdly, the study of ER affecting GTI as a complex and comprehensive subject is also a systematic social project. In-depth research in this field must open up new research areas and directions in order to produce quality research results. At the same time, cross-cutting and duplicative research as well as waste of research resources need to be avoided in order to promote substantial progress and breakthroughs in the field.

### 7.3. Limitations

This study has certain limitations. Firstly, this paper only retrieves and analyzes the literature of Science Citation Index Expanded (SCI-EXPANDED) and Social Sciences Citation Index (SSCI) in Web of Science and does not analyze the literature of other databases. Secondly, the setting of retrieval items is not comprehensive, which may miss some literature. Therefore, this paper is imperfect in data information collection, which will eventually have a certain impact on the research results.

### 7.4. Future Study

Firstly, the field of research on the impact of ER on GTI should be further broadened. Current research focuses on the impact of ER on GTI in the context of industry heterogeneity as well as regional heterogeneity, different types of environmental regulation, and different types of green technology innovation. Currently, the digital economy, as a new form of economic and social development, is increasingly prominent in optimizing the economic structure and promoting industrial transformation and upgrading. Little research has examined the combination of the digital economy with ER and GTI. In terms of literature combining the digital economy with ER and the digital economy with GTI, relevant research also needs to be increased. Future research could incorporate the digital economy into the research framework of this area in order to explore the new connotations of ER and GTI, their relationship and the mechanisms of influence in the context of the digital economy.

Secondly, existing studies have mainly focused on the relationship between different types of environmental regulation and different types of green technology innovation and the synergy between environmental regulation policies and R&D subsidies. However, less research has been conducted on the synergistic effects of different types of environmental regulations. Therefore, in the future, the synergistic effects formed by different types of environmental regulations and their impact on green technology innovation can be studied to better provide suggestions for the formulation of environmental regulation policies in different regions and industries.

Thirdly, future research can expand the scope of literature retrieval. The setting of retrieval terms should strive to be comprehensive. Comprehensive research methods can also be used to dig the hidden information in the data from multiple perspectives, so as to make the research results more realistic and objective.

## Figures and Tables

**Table 1:** Research data retrieval process in Web of Science.

Retrieval Setting Subjects	Retrieve Settings and Results
Database	Science Citation Index Expanded (SCI-EXPANDED) Social Sciences Citation Index (SSCI)
Retrieval mode	TS = (environmental regulation or environmental policy) and (green technology innovation or green patent)
Type of literature	Article, Review
Language type	English
Time span	2001–2021
Retrieval results	738 papers (695 articles and 43 reviews)

**Table 2:** Number of papers published by TOP 10 authors.

Item	Author	Frequency	Item	Author	Frequency
1	Massimiliano Mazzanti	9	6	Francesco Quatrao	5
2	Tobias Stucki	8	7	Hui Li	5
3	Francesco Nicolli	7	8	Claudia Chisetti	5
4	Martin Woerter	7	9	Herui Cui	4
5	Shi Yin	5	10	Annkristin Bercquist	4

**Table 3:** Frequency of TOP 12 co-cited authors.

Rank	Author	Frequency	Centrality	Rank	Author	Frequency	Centrality
1	Porter ME	277	0.12	7	OECD	119	0.06
2	Jaffe AB	197	0.05	8	Ambec S	106	0.09
3	Horbach J	147	0.04	9	Johnstone N	100	0.03
4	Popp D	146	0.04	10	Brunnermeier SB	97	0.14
5	Anonymous	131	0.04	11	Kemp R	97	0.02
6	Rennings K	128	0.02	12	Acemoglu D	88	0.06

**Table 4:** Number of papers issued by TOP 10 research institutions.

Item	Research Institution	Frequency	Item	Research Institution	Frequency
1	Harbin Engn Univ	18	6	Nanjing Univ Aeronaut & Astronaut	8
2	Univ Ferrara	15	7	Changan Univ	8
3	Jiangsu Univ	12	8	Xiamen Univ	8
4	Shanghai Univ Finance &Econ	11	9	Shandong Univ	8
5	SEEDS	10	10	Beijing Inst Technol	8

**Table 5:** Number of papers from TOP 12 countries (regions).

Rank	Country (Region)	Frequency	Centrality	Rank	Country (Region)	Frequency	Centrality
1	Peoples R China	301	0.25	7	France	34	0.16
2	Italy	72	0.15	8	Australia	27	0.06
3	USA	71	0.24	9	Netherlands	19	0.02
4	England	68	0.29	10	Switzerlands	18	0.02
5	Germany	37	0.02	11	South Korea	17	0.00
6	Spain	34	0.09	12	Canada	17	0.00

**Table 6:** Top 10 co-cited journals.

Rank	Cited Journal	Frequency	Centrality	Impact Factor
1	Journal of Cleaner Production	489	0.11	11.072
2	Ecological Economics	398	0.09	6.536
3	Energy Policy	381	0.06	7.576
4	Research Policy	371	0.02	9.473
5	Journal of Environmental Economics and Management	302	0.02	5.840
6	Technological Forecasting and Social Change	279	0.04	10.884
7	Environmental & Resource Economics	236	0.04	4.955
8	American Economic Review	233	0.17	11.490
9	Journal of Economic Perspectives	230	0.01	9.944
10	Energy Economics	226	0.02	9.252

**Table 7:** Frequency and centrality of TOP 16 keywords.

Item	Keyword	Frequency	Centrality	Item	Keyword	Frequency	Centrality
1	innovation	165	0.15	9	eco innovation	81	0.03
2	impact	165	0.05	10	empirical evidence	79	0.01
3	technology	149	0.07	11	green	76	0.05
4	performance	141	0.01	12	environmental regulation	66	0.02
5	policy	139	0.08	13	productivity	63	0.05
6	determinant	94	0.04	14	energy	60	0.11
7	research and development	92	0.04	15	environmental innovation	55	0.03
8	management	83	0.03	16	china	50	0.01

## Data Availability

The datasets used and analyzed during the current study are available from the corresponding author on reasonable request.
